# Comparative Efficacy of Acupuncture, Pharmacological Interventions, Sham Acupuncture, and Placebo for Migraine Prevention: A Bayesian Network Meta‐Analysis

**DOI:** 10.1155/prm/7778112

**Published:** 2026-09-20

**Authors:** Qian Zhu, Yidan Wang, Xinming Yang, Jin Yang, Xiaoli Song, Menghan Li, Lei Shi

**Affiliations:** ^1^ First Teaching Hospital of Tianjin University of Traditional Chinese Medicine, Tianjin, China, tjtcm.cn; ^2^ National Clinical Research Center for Chinese Medicine Acupuncture and Moxibustion, Tianjin, China, tjtcm.cn; ^3^ Tianjin University of Traditional Chinese Medicine, Tianjin, China, tjutcm.edu.cn; ^4^ Zhejiang Chinese Medical University, Hangzhou, China, zcmu.edu.cn; ^5^ The Third Affiliated Hospital of Zhejiang Chinese Medical University, Hangzhou, China, zjztyy.com; ^6^ Jinjiang Hospital of Traditional Chinese Medicine, Quanzhou, China

**Keywords:** acupuncture, Bayesian network meta-analysis, migraine prophylaxis, placebo

## Abstract

**Objective:**

This study aimed to compare the preventive effects of acupuncture (ACU), pharmacological interventions (PIs), sham ACU, and placebo for migraine through a Bayesian network meta‐analysis and to evaluate the relative efficacy of different treatment approaches.

**Methods:**

By reviewing PubMed, Embase, MEDLINE, and Web of Science databases, 32 randomized controlled trials (RCTs) were included in this study. Bayesian network analyses were performed using Stata and R software to compare the effects of ACU, PI, sham ACU, and placebo on reducing headache frequency, reducing monthly migraine days, alleviating headache intensity, and decreasing the use of analgesics. Treatment effects were estimated using the Markov Chain Monte Carlo method, and model convergence and heterogeneity were evaluated. The results were presented as pairwise comparisons and forest plots.

**Results:**

A total of 32 RCTs were included. Network meta‐analysis evaluated four intervention categories: ACU, PI, sham ACU, and placebo. For monthly migraine attack frequency, ACU was significantly more effective than placebo (MD = −2.30, 95% CrI: −3.70 to −1.00), and PI also showed superiority over placebo (MD = −1.70, 95% CrI: −2.50 to −0.82), while no significant differences were observed between ACU and PI or sham ACU. For monthly migraine days, both ACU (MD = −1.78, 95% CrI: −2.89 to −0.70) and PI (MD = −1.72, 95% CrI: −2.29 to −1.16) were superior to placebo, with no significant difference between ACU and PI (MD = −0.07, 95% CrI: −1.00 to 0.87). ACU demonstrated greater effects than PI in reducing headache intensity (MD = −0.99, 95% CrI: −1.94 to −0.02) and analgesic use frequency (MD = −1.12, 95% CrI: −1.98 to −0.08). However, ACU did not significantly differ from sham ACU across evaluated outcomes. Ranking analysis consistently indicated that ACU had the highest probability of being the most effective intervention, whereas placebo ranked lowest. These findings suggest that ACU may provide clinically meaningful benefits for migraine prevention, while the measurable effects observed in sham ACU groups highlight the methodological challenges of using sham ACU as an inert placebo control.

**Conclusion:**

This Bayesian network meta‐analysis suggests that ACU is effective for migraine prevention, showing significant benefits compared with placebo and comparable efficacy to PI. However, the lack of significant differences between ACU and sham ACU highlights the methodological challenges of sham ACU as a control intervention. Future studies should optimize sham ACU design to better evaluate the specific effects of ACU.

## 1. Background

Migraine is a common disabling primary headache disorder, characterized by recurrent, moderate to severe unilateral pulsating pain, often accompanied by photophobia, phonophobia, and gastrointestinal symptoms [1]. It ranks as the sixth most common disease and the second leading cause of disability worldwide [2], affecting approximately 1.04 billion people globally [3]. Migraine poses significant harm to both the physical and mental health of patients. Numerous studies have shown that migraine sufferers have a significantly higher incidence of cardiovascular diseases, such as ischemic stroke, coronary heart disease, and angina [4, 5], along with cognitive impairment [6, 7], and are more prone to anxiety and depression compared to the general population [8, 9]. Surveys indicate that around 38% of migraine patients could benefit from preventive treatment, with notable improvements in reducing headache days, severity, and the need for analgesics. However, only 3%–13% of patients actually receive preventive treatment in clinical practice [10]. Current preventive medications for migraines include antiepileptics (e.g., topiramate and valproate), calcium channel blockers (e.g., flunarizine), antidepressants (e.g., amitriptyline), and certain antihypertensive pharmacological interventions (PIs) (e.g., *β*‐blockers and angiotensin II receptor blockers such as candesartan), as well as newer PIs like monoclonal antibodies targeting CGRP and its receptor (e.g., erenumab and fremanezumab) [11]. These medications have solid evidence‐based support and are recommended in treatment guidelines.

Acupuncture (ACU) is commonly used as a preventive treatment for migraines due to its cost‐effectiveness, minimal side effects, and significant efficacy, making it widely adopted in clinical practice. With the advancement of evidence‐based medicine, randomized controlled trials (RCTs) have become the gold standard for verifying the efficacy and safety of treatments. ACU has often been compared with medications such as topiramate, valproate, or fremanezumab, as well as traditional Chinese medicine, and has demonstrated certain advantages in migraine prevention [12–14]. Zheng’s study found that ACU’s preventive effects were comparable to fremanezumab, but with a lower incidence of adverse events [12]. In a single‐blind RCT by Liu et al., ACU was shown to be superior to topiramate in reducing monthly headache days and alleviating patient anxiety [14]. Song et al. conducted an evidence‐based analysis of 40 RCTs involving 4405 patients. They found that manual ACU was superior to electroacupuncture and calcium channel blockers, indicating that ACU holds potential in migraine prevention [15].

Sham ACU is also commonly used in control groups, with various forms of sham ACU employed. These include specific, nonspecific, or nonacupoint sites, with nonacupoint sites being more commonly used. Sham ACU can also be classified by needle depth, such as superficial or deep needling, with superficial needling being more frequently applied. Other methods include blunt needles, mock electrical stimulation devices, and placebo laser ACU. In trials, different forms of sham ACU are combined to form a control group for comparison with true ACU. However, no sham ACU method fully meets the standards of a placebo control. Some RCTs comparing true and sham ACU [16–19] have shown no statistically significant differences, with both groups reducing the frequency, duration, and intensity of migraine attacks. This has led some researchers to speculate that ACU’s preventive effect on migraines may largely be a placebo effect. Due to the controversy surrounding sham ACU methods and the lack of accurate methods to verify ACU’s preventive effects on migraines, this study aims to compare ACU with guideline‐recommended medications to indirectly infer the efficacy and safety of ACU in migraine prevention.

## 2. Methods

### 2.1. Search Methods

Based on the research objective of evaluating the placebo effects of ACU and pharmacological therapies in migraine prevention, we conducted a preliminary database search to summarize relevant comparisons between ACU and PI for migraine prophylaxis. On the basis of this preliminary search, a comprehensive search strategy was developed. Search terms included keywords such as “prevent”, “acupuncture”, “migraine”, “randomized controlled trial”, “placebo”, “sham acupuncture”, “venlafaxine”, “flunarizine”, “metoprolol”, “valproic acid”, and “calcitonin gene‐related peptide”, together with their related controlled vocabulary terms, free‐text terms, synonyms, and upper‐ and lower‐level terms. The databases searched included PubMed, Web of Science, EMBASE, and MEDLINE, from database inception to July 25, 2024. Two researchers independently conducted the literature search and screening in parallel. All peer‐reviewed, officially published studies meeting the eligibility criteria were included. The detailed search strategy is available in Supporting Information Table 1.

**TABLE 1:** Characteristics of the included studies.

Included studies	Mean age/years		Sample size (M/F)		Mean disease duration/year		Interventions		Duration/week	Outcome measures
	T	C	T	C	T	C	T	C		
Allais [22]	38.4 ± 9.7	37.2 ± 9.3	0/77	0/73	NA	NA	ACU	Flunarizine	6 mon	①④
Amanat [23]	10.4 ± 2.8/11.2 ± 2.9	11.2 ± 3.1	63/43	27/25	NA	NA	Cinnarizine/Sodium valproate	Placebo	12	①③
Ashina [24]	41.3 ± 10.9	NA	80/303	NA	20.9 ± 11.9	NA	Erenumab 140 mg	NA	12	②
Ashrafi [25]	10.7 ± 2.4	8.9 ± 1.9	16/14	11/21	NA	NA	Flunarizine	Placebo	12	①③
Bigal [26]	40.7 ± 12.6	42.0 ± 11.6	15/82	12/92	16.9 ± 12.3	21.1 ± 14.1	TEV‐48125 675 mg	Placebo	12	②
Biçer [27]	33.76 ± 7.82	32.04 ± 9.13	5/24	4/21	NA	NA	ACU	Venlafa‐xine	3 mon	①②③
Dodick [28]	38.6 ± 10.8	39.0 ± 9.6	14/67	16/66	NA	NA	ALD403 1000 mg	Placebo	12	①②③
Ebrahimi‐Monfareda [29]	38.9 ± 9.2	38.9 ± 9.2	35	35	7.4 ± 1.2	7.4 ± 1.2	Valproic acid	Placebo	8	①③④
Ferrari [30]	45.9 ± 11.1	46.8 ± 11.1	45/238	46/233	24.0 ± 13.7	24.3 ± 13.6	Fremanezumab monthly	Placebo	12	②
Foroughipour [31]	35.8 ± 10.9	37.2 ± 11.2	21/29	20/30	NA	NA	ACU	Sham ACU	4	①
Giannini [32]	33.6 ± 17.4	34.7 ± 16.5	11/58	10/56	23.9 ± 10.0	24.3 ± 9.8	ACU	Drug	4 mon	①②④
Goadsby [33]	39.7 ± 11.9	40.5 ± 11.7	8/83	32/154	18.7 ± 12.5	20.6 ± 11.7	Atogepant 60 mg bid	Placebo	12	④
Kangasniemi [34]	37.5	37.5	15/59	15/59	17.2	17.2	Metoprolol	Placebo	8	①
Li [18]	36.8 ± 13.0	37.5 ± 12.1	67/291	15/103	104.0 ± 100.7 mon	102.0 ± 93.4 mon	ACU	Sham ACU	4	①②③
Linde [35]	35.2 ± 7.5	37.4 ± 8.6	0/15	0/13	NA	NA	ACU	Sham ACU	3 mon	①②③④
Mathew [36]	47	43	14/56	10/27	NA	NA	Divalproex	Placebo	12	①
Musil [37]	45.6 ± 12.8	46.5 ± 10.3	5/37	5/39	26.9 ± 12.9	23.0 ± 14.1	ACU	Drug	12	①③④
Sadeghian [38]	35.3	35.3	59	26	NA	NA	Valproate/L‐evetiracetam	Placebo	6 mon	①
Silberstein [39]	40.6 ± 12.0	41.4 ± 12.0	49/330	45/330	20.1 ± 12.0	19.9 ± 12.9	Fremanezumab monthly	Placebo	12	②
Sorge [40]	10.58 ± 3.25	10.70 ± 3.29	10/14	11/13	10.70 ± 3.29	10.58 ± 3.25	Flunarizine	Placebo	3 mon	①
Stauffer [41]	39.1 ± 11.5	41.3 ± 11.4	37/175	71/362	19.3 ± 11.9	19.9 ± 12.3	Galcanezumab 240 mg	Placebo	24	②
Streng [42]	40.0 ± 11.4	40.3 ± 10.7	7/52	6/48	14.5 ± 9.9	17.3 ± 10.8	ACU	Metoprolol	12	①②③④
Tao [43]	42 ± 13	42 ± 12	3/28	10/23	11.87 ± 8.28	12.36 ± 9.38	ACU	Metoprolol	8	①②③
Tfelt‐Hansen [44]	39.5 ± 9.9	39.5 ± 9.9	25/71	25/71	20.9 ± 10.7	20.9 ± 10.7	Timolol/Propr‐anolol	Placebo	12	①
Van de Ven [45]	38.3/38.9	38.9	29/122	11/64	NA	NA	Bisoprolol	Placebo	12	①
Wang [46]	31 ± 10	33 ± 9	0/32	0/32	11.8 ± 3.8 mon	12.7 ± 7.9 mon	ACU	Flunarizine	3 mon	①③
Wang [47]	37.1 ± 9.6	38.0 ± 10.1	40/184	57/281	11.2 ± 9.7	12.6 ± 10.2	Erenumab 140 mg	Placebo	12	②④
Winner [48]	40.6 ± 12.0	41.4 ± 12.0	49/330	45/330	20.1 ± 12.0	19.9 ± 12.9	Fremanezumab monthly	Placebo	12	②
Xu [49]	36.6 ± 12.0	36.0 ± 10.9	13/47	10/50	10.0 (5.0–19.5)	10.0 (6.0–14.0)	ACU	Sham ACU	8	①②③
Yu [50]	41.4 ± 10.9	41.9 ± 10.9	62/217	41/237	18.2 ± 11.9	17.8 ± 11.4	Erenumab 70 mg	Placebo	12	②④
Zhang [51]	33.04 ± 6.43	35.30 ± 9.43	0/24	0/20	NA	NA	ACU	Sham ACU	3 mon	①③
Zhao [52]	36.4 ± 14.2	39.1 ± 14.6	18/65	17/63	115.7 ± 99.5 mon	113.0 ± 104.2 mon	ACU	Sham ACU	4	①②③

*Note:* ①Migraine frequency; ②Monthly migraine days; ③Migraine intensity; ④Number of analgesic. ACU, acupuncture. Sham ACU, Sham acupuncture.

Abbreviation: NA, not available.

### 2.2. Inclusion and Exclusion Criteria

The included study types were RCTs. Non‐RCTs were excluded, such as quasirandomized trials, animal studies, systematic reviews, cohort studies, case reports, research abstracts, and dissertations. Patients were diagnosed with migraine, meeting the diagnostic criteria of the International Headache Society (IHS) for migraine [20]. There were no restrictions on patients’ age, gender, or severity of condition. Secondary migraines were excluded, such as those caused by tumors or other factors. Interventions included studies comparing ACU with sham ACU, ACU with medication, and prophylactic PI with placebos. Studies unrelated to the network chains were excluded. The primary outcome measure was the frequency of headache episodes per month. Secondary outcomes included monthly migraine days, headache intensity, and analgesic use frequency. Studies that did not include the primary and secondary outcome measures were excluded.

### 2.3. Literature Screening and Data Extraction

Literature was managed using EndNote X9 for deduplication. Two researchers independently screened records against predetermined inclusion/exclusion criteria. Disagreements were resolved through discussion with a third researcher. Using a predefined form, both researchers extracted data including: first author, publication year, country, publication type, corresponding author contact, experimental and control interventions, intervention duration, participant characteristics, group sample sizes, outcome measures, follow‐up duration, and outcome data. Authors were contacted for missing information. Extracted data underwent cross‐verification to ensure accuracy.

### 2.4. Risk of Bias Assessment

This study strictly adhered to the Cochrane Collaboration’s Handbook for Systematic Reviews of Interventions (Version 5.1.0) for assessing the risk of bias. Two researchers trained in evidence‐based medicine performed this evaluation independently, following strict inclusion and exclusion criteria. The key assessment areas include study design and randomization, allocation concealment, blinding, data completeness, selective reporting, and other sources of bias. Assessments are categorized into three levels: low, high, and unclear. Each assessor must record detailed reasons for their evaluations in each domain, and in cases of disagreement, a third researcher may be invited to participate and help reach a consensus.

### 2.5. Statistical Methods

Changes in all continuous variables were described using the mean (± standard deviation [SD]) with 95% confidence intervals (CIs), and statistical significance was assessed using *p* values. Network graphs and contribution plots were generated using Stata15 software. Bayesian network meta‐analyses (NMA) were performed using the gemtc package in R software Version 4.4.1 (available at https://cran.r-project.org/web/packages/gemtc/index.html), employing a random effects model to estimate both direct and indirect treatment effects [21]. Bayesian inference was conducted using the Markov Chain Monte Carlo (MCMC) method, with 50,000 iterations, the first 10,000 of which were burn‐in. Model convergence was assessed, and the final results were presented as pairwise comparisons, ranking probabilities, and forest plots. Between‐study heterogeneity was evaluated using *τ* ^2^ and *I* ^2^ statistics. Bayesian model convergence was assessed using PSRF and multivariate PSRF, and model fit was evaluated using Dbar, pD, and DIC.

## 3. Result

### 3.1. Characteristics of Included Studies

**FIGURE 1:**
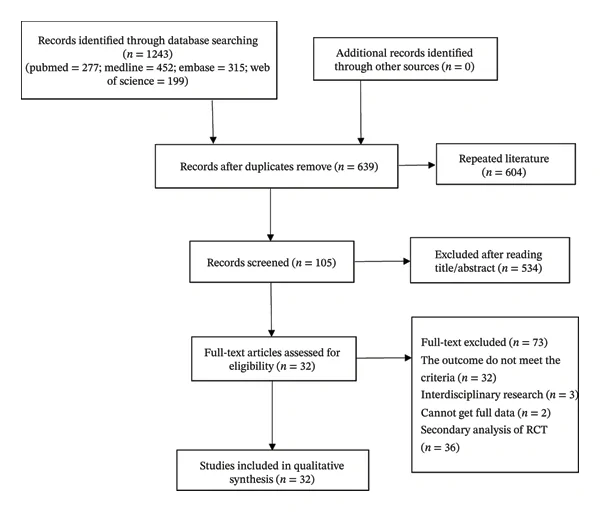
Literature screening process.

A total of 32 RCTs were included in this study [18, 22–52] (Figure 1), with detailed information on individual studies provided in the characteristics of the included studies (Table 1). This review covers RCTs conducted globally between 1984 and 2024, spanning three continents, including Europe, America, and Asia. All studies employed controlled designs with both experimental and control groups. The duration of treatment ranged from 4 weeks to 6 months. The largest selected comparison was reported by Silberstein 2017 [39], with 754 participants across the active treatment and control groups. Four studies specifically focused on female patients [22, 35, 46, 51], three studies concentrated on pediatric migraine patients [23, 25, 40], and the remaining studies involved adult migraine patients without gender restrictions. Most studies enrolled participants according to the IHS or International Classification of Headache Disorders diagnostic criteria [18, 22–30, 32, 33, 35–44, 46–52]. The remaining studies specified that participants were professionally diagnosed with migraine [31, 34], with one study further refining the inclusion criteria to patients with a history of 2 to 6 typical migraine attacks per month [45]. Thirteen studies evaluated the efficacy of ACU therapy [18, 22, 27, 31, 32, 35, 37, 42, 43, 46, 49, 51, 52]; five studies assessed calcium channel blockers, including flunarizine or cinnarizine [22, 23, 25, 40, 46]; four studies evaluated antiepileptic PI in the valproate class [23, 29, 36, 38], among which one study also assessed levetiracetam [38]; five studies evaluated *β*‐blockers, including bisoprolol, metoprolol, timolol, and propranolol [34, 42–45]; and one study assessed the atypical antidepressant venlafaxine [27]. In addition, nine studies evaluated calcitonin gene‐related peptide pathway monoclonal antibodies, including erenumab, fremanezumab, galcanezumab, and ALD403 [24, 26, 28, 30, 39, 41, 47, 48, 50], and one study assessed the oral CGRP receptor antagonist atogepant [33].

### 3.2. Risk of Bias in Included Studies

**FIGURE 2:**
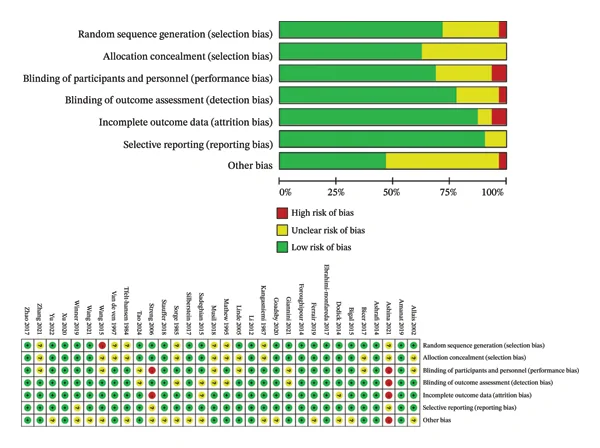
Risk of bias graph and summary.

This study assessed the risk of bias in the 32 included RCTs according to the Cochrane Collaboration’s Handbook for Systematic Reviews of Interventions, Version 5.1.0. The overall risk of bias is presented in Figure 1, and study‐specific judgments for each domain are summarized in Figure 2.

For random sequence generation, 23 studies were judged as low risk because they reported appropriate randomization methods [18, 22, 23, 25–33, 35, 38, 39, 41–43, 47–50, 52], eight studies were assessed as unclear risk due to insufficient details [24, 34, 36, 37, 40, 44, 45, 51], and one study was judged as high risk because participants were allocated according to visit order [46]. For allocation concealment, 20 studies reported adequate concealment methods and were assessed as low risk [18, 23, 25, 26, 28–33, 38, 39, 41–43, 47–50, 52], whereas 12 studies were judged as unclear risk [22, 24, 27, 34–37, 40, 44–46, 51]. Regarding blinding of participants and personnel, 22 studies were assessed as low risk [18, 23, 25, 26, 28–31, 33, 34, 36, 38–41, 44, 45, 47–50, 52], eight as unclear risk [22, 27, 32, 35, 37, 43, 46, 51], and two as high risk because of lack of blinding or open‐label design [24, 42]. For blinding of outcome assessment, 25 studies were judged as low risk [18, 22, 23, 25–31, 33–35, 39, 41, 42, 44–52], six as unclear risk [32, 36–38, 40, 43], and one as high risk [24]. For incomplete outcome data, 28 studies were assessed as low risk [18, 22, 23, 25–27, 29–37, 39–41, 43–52], two as unclear risk [28, 38], and two as high risk because of substantial or imbalanced attrition [24, 42]. For selective reporting, 29 studies were judged as low risk [18, 22, 23, 25–41, 43–47, 49–52], while three were assessed as unclear risk [24, 42, 48]. For other sources of bias, 15 studies were assessed as low risk [18, 23, 25, 27, 29, 31, 32, 35–37, 43, 45, 49, 51, 52], 16 as unclear risk [22, 26, 28, 30, 33, 34, 38–42, 44, 46–48, 50], and one as high risk [24].

## 4. Network Meta‐Analysis

### 4.1. Monthly Frequency of Migraine Attacks

**FIGURE 3:**
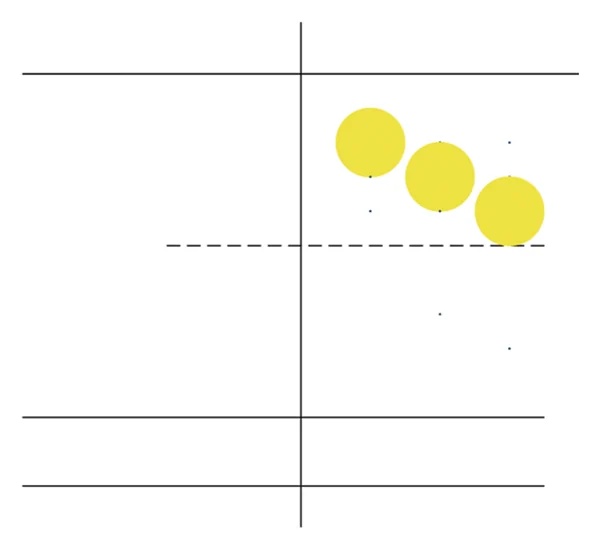
Network evidence graph and netweight graph. Notes: (A) network evidence graph; (B) netweight graph.

**FIGURE 4:**
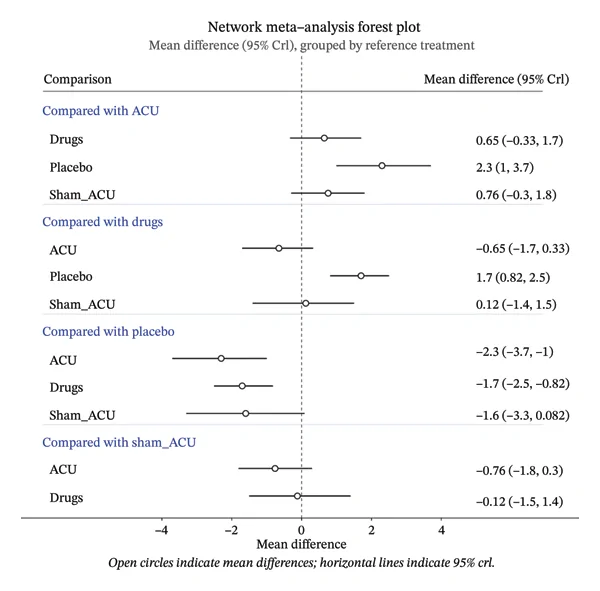
Mixed‐effects forest maps pairwise.

**FIGURE 5:**
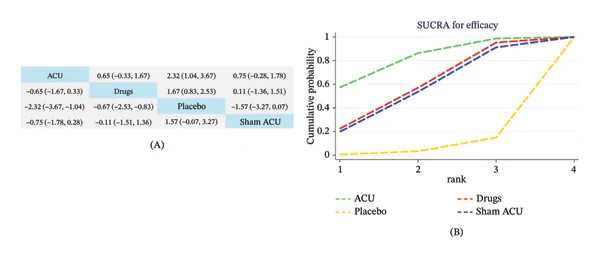
Pairwise comparison of inverted triangle plot and cumulative therapeutic effect SUCRA. Notes: (A) pairwise comparison of inverted triangle plot, (B) SUCRA for efficacy.

**FIGURE 6:**
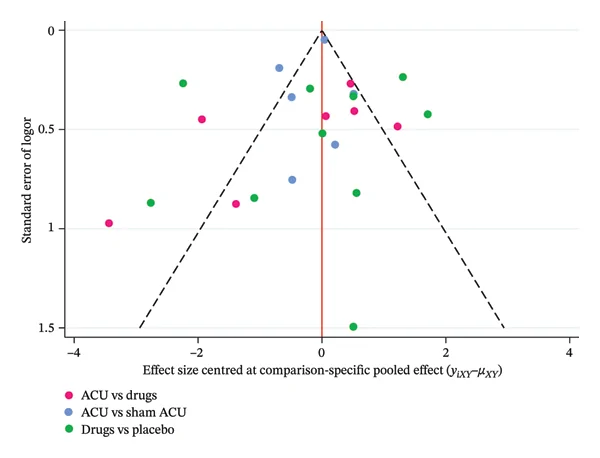
Funnel plot.

We included 23 studies in which both arms contained the primary outcome measure of monthly migraine attack frequency. Four intervention categories were analyzed: ACU, PI, placebo, and sham ACU. The PI group included conventional preventive PI and CGRP‐pathway treatments, including flunarizine, sodium valproate, venlafaxine, *β*‐blockers, levetiracetam, erenumab, fremanezumab, galcanezumab, ALD403, and atogepant. The network evidence graph showed that the comparison between PI and placebo contributed the largest amount of evidence, followed by ACU versus PI and ACU versus sham ACU (Figure 3A). In the netweight graph, the direct comparisons of ACU versus PI, ACU versus sham ACU, and PI versus placebo contributed 100% to their corresponding mixed estimates, with ACU versus PI carrying the greatest weight in the overall network (40%) (Figure 3B). A Bayesian random‐effects NMA showed good convergence, with all potential scale reduction factors close to 1 and a multivariate PSRF of 1. Model fit was acceptable, with a residual deviance (Dbar) of 46.7596, pD of 42.4656, and DIC of 89.2252. The between‐study SD was 1.20, and heterogeneity was substantial, with a global *I* ^2^ of 89.0%. The forest plot showed that ACU was significantly more effective than placebo in reducing monthly migraine attack frequency (MD = −2.30, 95% CrI: −3.70 to −1.00), while PI were also superior to placebo (MD = −1.70, 95% CrI: −2.50 to −0.82). No significant differences were observed between ACU and PI (MD = −0.65, 95% CrI: −1.70 to 0.33), ACU and sham ACU (MD = −0.76, 95% CrI: −1.80 to 0.30), or PI and sham ACU (MD = −0.12, 95% CrI: −1.50 to 1.40) (Figure 4). The league table yielded consistent findings (Figure 5A). The ranking probability analysis indicated the following order of effectiveness: ACU > PI≈sham ACU > placebo. ACU had the highest probability of ranking first (84.2%); sham ACU and PI showed comparable ranking probabilities in the middle ranks, while placebo was most likely to rank last, with a 97.0% probability of ranking fourth (Figure 5B). The comparison‐adjusted funnel plot was generally symmetrical, indicating no obvious small‐study effects or publication bias (Figure 6).

## 5. Monthly Migraine Days

Eighteen studies reporting monthly migraine days were included in the NMA. The network included 18 two‐arm studies, with 8 studies involving ACU, 14 involving PI, 10 involving placebo, and 4 involving sham ACU. Direct evidence was available for three comparisons: ACU versus PI, ACU versus sham ACU, and PI versus placebo. A Bayesian random‐effects model was used for the analysis. The model showed good convergence, with all potential scale reduction factors close to 1 and a multivariate PSRF of 1. Model fit was acceptable, with a residual deviance (Dbar) of 35.2009, pD of 30.8498, and DIC of 66.0507. The between‐study SD was 0.72. Heterogeneity was low for ACU versus PI (*I* ^2^ = 0.0%) but substantial for ACU versus sham ACU (*I* ^2^ = 76.1%) and PI versus placebo (*I* ^2^ = 79.3%); the global *I* ^2^ was 74.5%. The NMA showed that ACU was significantly superior to placebo in reducing monthly migraine days (MD = −1.78, 95% CrI: −2.89 to −0.70), and PI was also significantly superior to placebo (MD = −1.72, 95% CrI: −2.29 to −1.16). No significant differences were observed between ACU and PI (MD = −0.07, 95% CrI: −1.00 to 0.87), ACU and sham ACU (MD = −0.78, 95% CrI: −1.65 to 0.20), or PI and sham ACU (MD = −0.71, 95% CrI: −1.99 to 0.63). The ranking probability analysis indicated the following order of effectiveness: ACU≈PI > sham ACU > placebo.

### 5.1. Headache Intensity

Fourteen studies reporting headache intensity were included in the NMA. The network included 14 two‐arm studies, with 10 studies involving ACU, 9 involving PI, 4 involving placebo, and 5 involving sham ACU. Direct evidence was available for ACU versus PI, ACU versus sham ACU, and PI versus placebo. A Bayesian random‐effects model showed good convergence, with all potential scale reduction factors close to 1 and a multivariate PSRF of 1. Model fit was acceptable, with a residual deviance (Dbar) of 28.4207, pD of 26.5437, and DIC of 54.9644. The between‐study SD was 0.96, and the residual heterogeneity was low, with an *I* ^2^ of 5%. The NMA showed that ACU was significantly superior to PI in reducing headache intensity (MD = −0.99, 95% CrI: −1.94 to −0.02) and significantly superior to placebo (MD = −2.27, 95% CrI: −3.73 to −0.86). PI was also significantly superior to placebo (MD = −1.28, 95% CrI: −2.37 to −0.24). However, no significant differences were observed between ACU and sham ACU (MD = −0.84, 95% CrI: −1.80 to 0.10), PI and sham ACU (MD = 0.15, 95% CrI: −1.24 to 1.49), or sham ACU and placebo (MD = −1.43, 95% CrI: −3.15 to 0.30). The ranking probability analysis indicated the following order of effectiveness: ACU > sham ACU > PI > placebo.

### 5.2. Analgesic Use Frequency

Ten studies reporting analgesic use frequency were included in the NMA. The network included 10 two‐arm studies, with 6 studies involving ACU, 9 involving PI, 4 involving placebo, and 1 involving sham ACU. Direct evidence was available for ACU versus PI, ACU versus sham ACU, and PI versus placebo. A Bayesian random‐effects model showed good convergence, with all potential scale reduction factors close to 1 and a multivariate PSRF of 1. Model fit was acceptable, with a residual deviance (Dbar) of 19.9378, pD of 17.4322, and DIC of 37.3699. The between‐study SD was 0.73, and the residual heterogeneity was low, with an *I* ^2^ of 5%. In direct pairwise comparisons, heterogeneity was substantial for ACU versus PI (*I* ^2^ = 76.8%) and moderate for PI versus placebo (*I* ^2^ = 45.8%). The NMA showed that ACU was significantly superior to PI in reducing analgesic use frequency (MD = −1.12, 95% CrI: −1.98 to −0.08) and significantly superior to placebo (MD = −2.54, 95% CrI: −3.81 to −1.13). PI was also significantly superior to placebo (MD = −1.41, 95% CrI: −2.43 to −0.49). However, no significant differences were observed between ACU and sham ACU (MD = −0.51, 95% CrI: −3.64 to 2.68), PI and sham ACU (MD = 0.61, 95% CrI: −2.67 to 3.88), or sham ACU and placebo (MD = −2.03, 95% CrI: −5.46 to 1.42). The ranking probability analysis indicated the following order of effectiveness: ACU > sham ACU > PI > placebo.

## 6. Discussion

In this NMA, we explored the relative efficacy of various treatments in reducing monthly migraine frequency, alleviating headache intensity, and decreasing the frequency of analgesic use. The results indicate that the ACU group demonstrated superior efficacy in multiple areas. In comparisons of monthly migraine frequency, headache intensity, and analgesic use, ACU outperformed the PI group, although some indicators did not reach statistical significance. In contrast, the difference in efficacy between the ACU group and the placebo group was statistically significant, while the difference between the ACU group and the sham ACU group was not. SUCRA rankings showed that ACU ranked first among all treatment options, followed by the PI group and sham ACU group. Compared to the placebo group, ACU demonstrated significantly better efficacy, further supporting the potential benefits of ACU therapy.

ACU, a safe and feasible traditional Chinese therapy, is important for migraine prevention and treatment. This may occur via regulation of the trigeminovascular system and neurotransmitters, improved cerebral blood flow, and qi‐blood balance. Qu et al. found electroacupuncture significantly increased periorbital mechanical pain thresholds and reduced neuronal discharge in the trigeminal cervical complex, demonstrating migraine prevention in rats [53]. Liu et al.’s proteomic and metabolomic analyses revealed ACU reversed key molecular levels (e.g., FAD, L‐norepinephrine, *α*‐D‐glucose, BLVRB, and L‐glutamate) and induced systemic pathway changes related to immunity, arginine, and energy metabolism in migraine patients without aura [54]. Chang’s study showed real ACU, versus sham, normalized functional connectivity in networks including RVM/TCC, frontal‐parietal, and cingulate‐tegmental, while also normalizing sensory‐motor network connections to regions involved in sensation, emotion, and cognition. These neuroimaging findings confirm ACU prevents and treats migraines through central regulation [55].

While accumulating evidence supports the preventive effect of ACU for migraine, several clinical trials have reported no significant differences between real and sham ACU [17, 18, 56–58]. In the present study, sham ACU showed measurable effects and was not consistently inferior to active interventions, suggesting that sham ACU may not represent a completely inert placebo control. Increasing evidence indicates that different sham ACU techniques, including superficial needling, nonacupoint stimulation, and nonpenetrating placebo needles, may produce different levels of contextual and physiological effects, which could influence the estimation of ACU‐specific effects [59, 60]. Superficial or minimal needling may still stimulate peripheral sensory receptors and induce neurophysiological responses, while nonacupoint stimulation may activate pain‐modulating pathways, particularly in analgesic conditions [61]. Therefore, inserted sham ACU procedures may introduce specific effects rather than solely representing placebo effects, potentially leading to an underestimation of the true ACU effect [62]. The selection of an appropriate sham control is therefore crucial for future ACU trials. Validated nonpenetrating sham devices, such as Streitberger, Park, and Takakura‐type needles, have been developed to improve blinding while minimizing physiological stimulation; however, their validity and effectiveness require further assessment in different clinical populations [59]. Based on current methodological evidence, future migraine prevention trials should prioritize sham procedures that minimize needle penetration, avoid stimulation of recognized acupoints, reduce de qi‐related sensory input, and incorporate formal assessments of blinding credibility. Such standardized sham designs may improve the methodological rigor of ACU trials and allow more accurate estimation of the specific therapeutic effects of ACU.

This study has some limitations in exploring the efficacy of ACU, sham ACU, PI, and placebos in treating migraines. The inconsistency between different sham ACU and drug protocols increased heterogeneity among the groups, reducing the comparability of the results—one of the inherent limitations of Bayesian NMA. Small sample sizes limited the statistical power of some comparisons, and certain treatment effect estimates lacked precision. Moreover, the study primarily focused on short‐term efficacy, with a lack of long‐term follow‐up data, preventing a comprehensive evaluation of the sustainability and safety of treatments. While the Bayesian model showed good fit, it relies on prior assumptions, which may introduce subjectivity into the results. In addition, this review is that Chinese databases, such as the China National Knowledge Infrastructure (CNKI), Wanfang Database, VIP Database, and SinoMed, were not searched. Because a considerable proportion of ACU trials are conducted and published in China, the exclusion of Chinese‐language databases may have limited the comprehensiveness of the evidence base and introduced potential language or regional publication bias. Therefore, some relevant ACU studies published only in Chinese may not have been captured. Future reviews should include both international and Chinese databases to provide a more comprehensive assessment of ACU‐related evidence in migraine prevention. Lastly, publication bias was not fully assessed, potentially overestimating the efficacy of certain treatments. Future research should aim to improve sham ACU designs, increase sample sizes, reduce heterogeneity, and focus on long‐term efficacy and multidimensional clinical outcomes.

## 7. Conclusion

This NMA compared ACU, sham ACU, PI, and placebo for reducing monthly migraine frequency, monthly migraine days, headache intensity, and analgesic use. ACU demonstrated superior efficacy, particularly in reducing migraine attack frequency versus placebo, confirming its potential for migraine prevention/treatment. However, the lack of significant difference between ACU and sham ACU challenges sham ACU’s suitability as a placebo control. Literature suggests sham methods (superficial needling or nonacupoint stimulation) may elicit physiological responses, yielding effects similar to ACU and thus failing as an ideal placebo. Future studies should optimize sham design, increase sample sizes, extend follow‐up, and refine protocols to accurately assess ACU’s true efficacy for migraine.

## Funding

This work was supported by the Research Plan Project of Tianjin Municipal Education Commission, Tianjin University of Traditional Chinese Medicine (Grant No. 2025KJ117).

## Consent

The authors have nothing to report.

## Conflicts of Interest

The authors declare no conflicts of interest.

## Supporting Information

Additional supporting information can be found online in the Supporting Information section.

## Supporting information


**Supporting Information 1** Supporting Table 1 presents the detailed search strategy for PubMed.

**Supporting Information 2** PRISMA_2020_checklist.

## Data Availability

The data that support the findings of this study are available on request from the corresponding author. The data are not publicly available due to privacy or ethical restrictions.
