# Supplementary material for: Comparative Efficacy of Acupuncture, Pharmacological Interventions, Sham Acupuncture, and Placebo for Migraine Prevention: A Bayesian Network Meta‐Analysis
Source: Pain Res Manag. 2026 Sep 20;2026:7778112. doi: 10.1155/prm/7778112 (PMC13591117; doi:10.1155/prm/7778112)
Supplement: Supplementary file 1 [file PRM-2026-7778112-s001.docx]

Table 1. Search strategy for PubMed

#1	((Acupuncture [MeSH Terms]) OR (Acupuncture [MeSH Major Topic])) OR (Acupuncture[Title/Abstract] OR Acupoint[Title/Abstract] OR Electroacupuncture[Title/Abstract] OR Electro-acupuncture[Title/Abstract] OR Electric acupuncture[Title/Abstract])

#2	((Disorder, Migraine [MeSH Major Topic]) OR (Disorder, Migraine [MeSH Terms])) OR (Disorder, Migraine[Title/Abstract] OR Disorders, Migraine[Title/Abstract] OR Migraine Disorder[Title/Abstract] OR Headache, Migraine[Title/Abstract] OR Headaches, Migraine[Title/Abstract] OR Migraine Headaches[Title/Abstract] OR Migraine[Title/Abstract] OR Migraines[Title/Abstract] OR Migraine Headache[Title/Abstract] OR Acute Confusional Migraine[Title/Abstract] OR Acute Confusional Migraines[Title/Abstract] OR Migraine, Acute Confusional[Title/Abstract] OR Migraines, Acute Confusional[Title/Abstract] OR Status Migrainosus[Title/Abstract] OR Abdominal Migraine[Title/Abstract] OR Abdominal Migraines[Title/Abstract] OR Migraine, Abdominal[Title/Abstract] OR Migraines, Abdominal[Title/Abstract] OR Cervical Migraine Syndrome[Title/Abstract] OR Cervical Migraine Syndromes[Title/Abstract] OR Migraine Syndrome, Cervical[Title/Abstract] OR Migraine Syndromes, Cervical[Title/Abstract] OR Hemicrania Migraine[Title/Abstract] OR Hemicrania Migraines[Title/Abstract] OR Migraine, Hemicrania[Title/Abstract] OR Migraines, Hemicrania[Title/Abstract] OR Migraine Variant[Title/Abstract] OR Migraine Variants[Title/Abstract] OR Variant, Migraine[Title/Abstract] OR Variants, Migraine[Title/Abstract])

#3	((Randomized Controlled Trial [MeSH Major Topic]) OR (Randomized Controlled Trial [MeSH Terms])) OR (RCT[Title/Abstract] OR Randomized Controlled Trial[Title/Abstract] OR Controlled Clinical Trial[Title/Abstract] OR Randomized[Title/Abstract] OR Clinical Trials[Title/Abstract] OR Randomly[Title/Abstract] OR trial[Title/Abstract])

#4	(Prevent[Title/Abstract] OR Precaution[Title/Abstract] OR prophylaxis[Title/Abstract])

#5	#1 AND #2 AND #3 AND #4

#6	(review[Title/Abstract] OR meta[Title/Abstract])

#7	#5 NOT #6

#8	“Migraine frequency” or “attack frequency” or “migraine attack” or “frequency of migraine attack”

#9	#7 AND #8

#10 ((Hydrochloride, Venlafaxine OR Effexor OR Efexor OR Trevilor OR Vandral OR Venlafaxine OR Dobupal OR Sila Venlafaxine OR Flunarizin OR Sibelium OR Flunarizine Hydrochloride OR Hydrochloride, Flunarizine OR Flunarizine Dihydrochloride OR Dihydrochloride, Flunarizine OR Metoprolol OR Betaloc OR Spesicor OR Spesikor OR Metoprolol Tartrate OR Lopressor OR Betalok OR Beloc Duriles OR Metoprolol Succinate OR Seloken OR Toprol OR Sumatriptan OR Sumatriptan Succinate OR Imigran OR Triptan OR Valproic Acid OR Divalproex OR Valproate Sodium OR Sodium Valproate OR Valproate OR Dipropyl Acetate OR Magnesium Valproate OR Divalproex Sodium OR Semisodium Valproate OR Depakote OR Vupral OR Ergenyl OR Depakene OR Convulsofin OR Depakine OR Calcium Valproate OR Valproate Calcium OR Propylisopropylacetic Acid OR calcitonin gene-related peptide OR CGRP OR anti-CGRP OR CGRP inhibitor* OR CGRP antagonist* OR CGRP receptor antagonist* OR CGRP monoclonal antibod* OR anti-CGRP monoclonal antibod* OR CGRP-targeted therap* OR CGRP-pathway therap* OR gepants OR erenumab OR fremanezumab OR galcanezumab OR eptinezumab OR ALD403 OR TEV-48125 OR TEV48125 OR AMG 334 OR AMG334 OR LY2951742 OR atogepant OR rimegepant[MeSH Terms])) OR (Hydrochloride, Venlafaxine[Title/Abstract] OR Effexor[Title/Abstract] OR Efexor[Title/Abstract] OR Trevilor[Title/Abstract] OR Vandral[Title/Abstract] OR Venlafaxine[Title/Abstract] OR Dobupal[Title/Abstract] OR Sila Venlafaxine[Title/Abstract] OR Flunarizin[Title/Abstract] OR Sibelium[Title/Abstract] OR Flunarizine Hydrochloride[Title/Abstract] OR Hydrochloride, Flunarizine[Title/Abstract] OR Flunarizine Dihydrochloride[Title/Abstract] OR Dihydrochloride, Flunarizine[Title/Abstract] OR Metoprolol[Title/Abstract] OR Betaloc[Title/Abstract] OR Spesicor[Title/Abstract] OR Spesikor[Title/Abstract] OR Metoprolol Tartrate[Title/Abstract] OR Lopressor[Title/Abstract] OR Betalok[Title/Abstract] OR Beloc Duriles[Title/Abstract] OR Metoprolol Succinate[Title/Abstract] OR Seloken[Title/Abstract] OR Toprol[Title/Abstract] OR Sumatriptan[Title/Abstract] OR Sumatriptan Succinate[Title/Abstract] OR Imigran[Title/Abstract] OR Triptan[Title/Abstract] OR Valproic Acid[Title/Abstract] OR Divalproex[Title/Abstract] OR Valproate Sodium[Title/Abstract] OR Sodium Valproate[Title/Abstract] OR Valproate[Title/Abstract] OR Dipropyl Acetate[Title/Abstract] OR Magnesium Valproate[Title/Abstract] OR Divalproex Sodium[Title/Abstract] OR Semisodium Valproate[Title/Abstract] OR Depakote[Title/Abstract] OR Vupral[Title/Abstract] OR Ergenyl[Title/Abstract] OR Depakene[Title/Abstract] OR Convulsofin[Title/Abstract] OR Depakine[Title/Abstract] OR Calcium Valproate[Title/Abstract] OR Valproate Calcium[Title/Abstract] OR Propylisopropylacetic Acid[Title/Abstract] OR calcitonin gene-related peptide[Title/Abstract] OR CGRP[Title/Abstract] OR anti-CGRP[Title/Abstract] OR CGRP inhibitor*[Title/Abstract] OR CGRP antagonist*[Title/Abstract] OR CGRP receptor antagonist*[Title/Abstract] OR CGRP monoclonal antibod*[Title/Abstract] OR anti-CGRP monoclonal antibod*[Title/Abstract] OR CGRP-targeted therap*[Title/Abstract] OR CGRP-pathway therap*[Title/Abstract] OR gepants[Title/Abstract] OR erenumab[Title/Abstract] OR fremanezumab[Title/Abstract] OR galcanezumab[Title/Abstract] OR eptinezumab[Title/Abstract] OR ALD403[Title/Abstract] OR TEV-48125[Title/Abstract] OR TEV48125[Title/Abstract] OR AMG 334[Title/Abstract] OR AMG334[Title/Abstract] OR LY2951742[Title/Abstract] OR atogepant[Title/Abstract] OR rimegepant[Title/Abstract])

#11	#10 AND #2 AND #3 AND #4

#12	#11 NOT #6

#13	#12 AND #8

#14	#9 OR #13
